# Impact of *Globodera ellingtonae* on yield of potato (*Solanum tuberosum*)

**DOI:** 10.21307/jofnem-2019-073

**Published:** 2019-10-14

**Authors:** Inga A. Zasada, Russell E. Ingham, Hannah Baker, Wendy S. Phillips

**Affiliations:** 1 USDA-ARS Horticultural Crops Research Laboratory, Corvallis, OR, 97330; 2 Department of Botany and Plant Pathology, Oregon State University, Corvallis, OR, 97331

**Keywords:** Potato, Damage, Globodera, Regression

## Abstract

*Globodera ellingtonae* was described from Oregon and Idaho in 2012. Due to the close phylogenetic relationship of this nematode to the potato cyst nematodes *G. pallida* and *G. rostochiensis*, and evidence that *G. ellingtonae* reproduces on potato (*Solanum tuberosum*), potential damaging effects to potato by this nematode are of great concern. To evaluate the pathogenic effects of *G. ellingtonae* on potato, five field and two microplot trials were conducted over a four-year period including comparisons of a range of *G. ellingtonae* initial population densities (*Pi*) and potato cultivars. In two field trials, potato ‘Russet Burbank’ was inoculated with *Pi* of *G. ellingtonae* ranging from 0 to 80 eggs/g soil; a similar trial was conducted with potato ‘Désirée.’ In another field trial, potato cultivars varying in maturity lengths were either inoculated (80 eggs/g soil) or not with *G. ellingtonae*. In a final field trial, ‘Ranger Russet’ was inoculated with *Pi* of *G. ellingtonae* ranging from 0 to 360 eggs/g soil. Additionally, Russet Burbank was inoculated with *G. ellingtonae Pi* ranging from 0 to 169 eggs/g soil in microplots. In all trials, data on tuber yield, aboveground biomass, final eggs/cyst, final population densities (*Pf*), and reproduction factor (RF = *Pf/Pi*) were collected. In only two of six trials conducted with increasing levels of *Pi*, was there a significant negative correlation between *Pi* of *G. ellingtonae* and yield of potato. Based on the linear regression model of tuber yield on log*Pi* for Russet Burbank, 30.5 to 40.9% yield loss was predicted at a *Pi* of 40 and 80 eggs/g soil, respectively, and for Ranger Russet, 16.5 and 19.7% yield loss was predicted at a *Pi* of 40 and 80 eggs/g soil, respectively. None of the potato cultivars inoculated with 80 *G. ellingtonae* eggs/g soil had significantly reduced yields compared to non-inoculated plants. Reproduction factor values across trials ranged from 4.0 to 8.3 when inoculated with *Pi* of 40 eggs/g soil, demonstrating that the nematode successfully invaded and reproduced on potato in all trials. Care should be taken in extrapolating the results from these experiments conducted in Oregon to probable effects of *G. ellingtonae* on potato in other environments.KeywordsPotato, Damage, Globodera, Regression

The potato cyst nematodes (PCN) *Globodera pallida* (Stone) Behrens and *G. rostochiensis* (Wollenweber) Behrens occur worldwide and can cause over 80% yield loss of potato (*Solanum tubersoum*) in heavily infested fields (Brown, 1969; Greco and Moreno, 1992). Both species occur in the USA: *G. rostochiensis* was discovered in New York in 1941 and *G. pallida* was found in Idaho in 2006 (Chitwood et al., 1942; Hafez et al., 2007). Limiting the distribution of PCN is of highest importance to the US potato industry. As a result of the *G. pallida* find in Idaho, an extensive survey of potato-producing regions was undertaken in the USA to determine the origin and distribution of PCN. As part of this survey, additional potato acreage in the Pacific Northwest of the USA was sampled.

In one survey, in 2007, a morphologically distinct cyst nematode but very similar PCN was found at two locations in Idaho (Skantar et al., 2011). Independent of this survey, another unusual *Globodera* population was found at the Oregon State University Central Oregon Agricultural Research Center (OSU-COARC), Powell Butte, OR. These populations were both subsequently determined to be a new species: *G. ellingtonae* Handoo, Carter, Skantar, and Chitwood (Handoo et al., 2012). Phylogenetic analyses based on ITS rDNA places *G. ellingtonae* in a monophyletic clade containing both *G. pallida* and *G. rostochiensis* as well as the tobacco cyst nematode *G. tabacum* (Skantar et al., 2011). Due to the close phylogenetic relationship of *G. ellingtonae* to *G. pallida* and *G. rostochiensis* and evidence that *G. ellingtonae* reproduces on potato, potential damaging effects to potato by this nematode are of great concern (Skantar et al., 2011; Handoo et al., 2012; Zasada et al., 2013; Lax et al., 2014).

To address whether *G. ellingtonae* is a pathogen of potato, field research was undertaken at OSU-COARC, one of the original locations where this nematode was found. Prior to the discovery of *G. ellingtonae* at this research center, potatoes were grown on a four-year rotation with the field used for this research last cropped to potato in 2008. It is unknown when *G. ellingtonae* was introduced at this location. During the initial survey for *Globodera* sp. conducted in 2008, *G. ellingtonae* was found at very low densities (1-7 cysts/2.3 kg soil) across the farm, with one hotspot being identified with 24 cysts/2.3 kg soil. To enable the inclusion of a range of initial population densities, potato tubers were inoculated with cysts utilizing a strategy similar to that reported in the literature (Lamondia and Brodie, 1986; Smit and Vamerali, 1998). This strategy also allowed for the inclusion of higher initial population densities that would not have been achievable relying only on endemic field populations.

Because PCN generally have just one generation per year and become immobile after initially infecting a host as second-stage juveniles (J2), initial soil egg population density (*Pi*) is expected to correlate strongly with yield loss (Seinhorst, 1965; Oostenbrink, 1966). Considerable research has been conducted to correlate the *Pi* of PCN to end of season yield losses (Seinhorst, 1965, 1982; Oostenbrink, 1966; Brown, 1969; Elston et al., 1991; ). We experimentally varied *Pi* of *G. ellingtonae* and measured end of season potato yield, dry foliar weight, and final nematode population densities (*Pf*) in field and microplot experiments.

## Materials and methods

### Microplot experiment

Inoculum of *G. ellingtonae* was produced at the Oregon State University Central Oregon Agricultural Research Center (OSU-COARC), Powell Butte, OR by planting potato Russet Burbank into 22-liter pots buried in the soil containing *G. ellingtonae* cysts in May 2015. Plants were allowed to grow for four months, receiving irrigation and fertilizer. The pots were collected and soil was emptied onto a tarp, dried, and passed through a 4-mm sieve. The soil was mixed and subsamples (100 g) collected to determine *G. ellingtonae* initial population densities (*Pi)*. Cysts were extracted from ten 100 g soil subsamples using a USDA cyst extractor (Ayoub, 1980), collected and counted. The number of eggs/cysts was determined by crushing all the collected cysts within the subsample with a rubber stopper on a 60- over a 500-mesh sieve (Zasada et al., 2013). The eggs that were retained on the 500-mesh sieve were washed into a 50 ml polystyrene tube. Eggs were enumerated by counting two 1 ml aliquots using an inverted microscope. The average cyst density of the soil was 0.6 (±0.04) cysts/g soil, the average egg density was 169 (±13) eggs/g soil, and the average number of eggs/cyst in soil was 255 (±26). Soil without *G. ellingtonae* was collected on a part of the farm at Powell Butte, OR where no *G. ellingtonae* had been found. Collected soil was dried and passed through a 4-mm sieve. Both the infested and uninfested soil is a Redmond ashy sandy loam.

The experiment was conducted in 2017 in microplots in Corvallis, OR. The experimental unit was a 5-liter pot, and pots were placed into beds that were 2.4 × 1.2 × 0.3 m. The bed frames were constructed with wood and lined with pond liner. Soil containing *G. ellingtonae* was mixed with non-cyst-containing soil to achieve varying *Pi* (Table 1). The total weight of soil in the pots was 4,800 g. During the process of mixing the soils, water was added to the mixture to create an initially moist environment. The pots were placed into the beds and the remaining area around the pots was filled with soil to help insulate the pots. A single-drop potato Russet Burbank tuber was planted approximately 6 cm deep in each pot. Two trials were conducted each occupying two beds with the second trial established a week after the first trial. The experimental design was completely randomized for both trials with treatments replicated six times.

**Table 1.** Summary of microplot and field trials conducted to determine the pathogenicity of *Globodera ellingtonae* on potato (*Solanum tuberosum*).

Name	Year	Cultivar	Initial nematode density (eggs/g soil) evaluated
Microplot 1^a^	2017	Russet Burbank	0, 6, 12, 26, 52, 104, 169
Microplot 2	2017	Russet Burbank	0, 6, 12, 26, 52, 104, 169
Trial 1^b^	2013	Russet Burbank	0, 10, 20, 40, 80
Trial 2	2013	Désirée	0, 10, 20, 40, 80
Trial 3	2014	Russet Burbank	0, 10, 20, 40, 80
Trial 4	2014	Various varieties	0, 80
Trial 5	2015	Ranger Russet	0, 40, 80, 160, 320

^a^Microplot trials were conducted in Corvallis, OR; ^b^field trials were conducted in Powell Butte, OR.

The pots were watered daily or as needed. The plants were fertilized with Osmocote Smart-Release Plant Food (Scotts Miracle-Gro; Marysville, OH) at planting and then one more time approximately 3-weeks-after emergence with 20N-20P-20K fertilizer (Peters; Allentown, PA). Over the course of the experiments, any plant health issues (insect or pathogen damage, chlorosis, necrosis) were noted. After the majority of the plants within a trial had senesced, at 16 and 15 wk in trials 1 and 2, respectively, the trials were terminated. At this time, the tops of the plants were removed and placed into a 70°C dryer for a week and then weighed to obtain aboveground biomass. Each pot was then lifted out of the ground and the contents of the pot emptied into a container. The contents of the pot were laid out to dry for a week in a greenhouse. The tubers were collected and the soil was mixed and a 100 g subsample of soil collected for *G. ellingtonae* extraction as described above. The tubers were counted and each tuber was weighed individually.

A two-way analysis of variance (ANOVA) with trial, *Pi* and trial × *Pi* was used to test for difference in mean yields, aboveground biomass, tuber number, and individual tuber weight, followed by a Tukey’s honest significant difference (HSD) test for pairwise comparisons; since trial was not significant in the model, data from the trials were combined for presentation. To test for a relationship between *Pi* and tuber yield and *Pi* and aboveground biomass, linear regression with log_10_ transformed *Pi* (*x* + 1) was performed in concordance with the relationship between *Pi* and yield proposed by Oostenbrink (1966); data were combined from the trials for linear regressions. Although various models have been proposed to describe the relationship between *Pi* and yield for PCN, the loglinear equation of Oostenbrink (1966) was used as it is simpler, does not require estimation of unknown parameters such as minimum yield and damage threshold, and has been determined to fit as well or better than other proposed relationships at mid-range *Pi* (Mulder et al., 1997). Host status and reproductive ability were measured by eggs/cyst, final density of eggs/g soil (*Pf*), and reproduction factor (RF) calculated as *Pf*/*Pi*, with differences tested by two-way ANOVA and Tukey’s HSD. Data were transformed to log_10_ (*x* + 1) when necessary to meet the assumptions of ANOVA. All statistics were performed using JMP 13 (SAS Institute, Cary, NC).

### Field experiments

Field trials were carried out in 2013 (two trials), 2014 (two trials), and 2015 (one trial) at OSU-COARC, Powell Butte, OR (Table 1). The field used in all years and trials had been in a long-term four-year potato rotation. It was planted to barley (*Hordeum vulgare*) and Austrian winter pea (*Pisum sativum*) in 2007, to potato in 2008, and was fallow in 2009 and 2010. At the end of summer 2010, the field was planted to a winter oat (*Avena sativa*) cover crop and in all subsequent winters was cover cropped with winter wheat (*Triticum aerstivum*). Each year, trials were placed in an area of the field where pretreatment soil sampling did not recover *G. ellingtonae* cysts. The soil at Powell Butte, OR is a Redmond ashy sandy loam.

In each year, an area approximately 20-m wide × 45-m long was prepared for potato planting. Treatments of varying *Pi* (Table 1) were applied to individual potato plants using cysts reared as described above. In 2014, a trial was conducted to evaluate additional potato cultivars from different maturity classes two early (‘Russet Norkotah’ and ‘Yukon Gold’), one mid (‘Ranger Russet’), and two late (‘Umatilla Russet’ and ‘Alturas’) maturing cultivars were used. Yukon Gold is a yellow table stock cultivar, Désirée is a red table stock cultivar, and all of the other cultivars are russeted types used primarily in processing. In all trials, *Pi*/cultivar combinations were replicated at least seven times. All trials were arranged in a completely randomized block design in a single (Russet Burbank and Désirée trials) or multiple (cultivar and Ranger Russet trials) rows. Additional rows of potatoes were planted on either side of experimental rows to serve as an environmental buffer.

Prior to establishing treatments, the entire area was treated with the pre-emergence herbicide S-ethyl dipropylthiocarbamate (Eptam, Gowan Company, Yuma, AZ, USA) at a rate of 4.1 liter/ha and 1,344 kg/ha triple 16 (16-16-16 N-P-K) prior to shallow cultivation. To plant and inoculate potatoes, a 30-cm diam. × 30-cm deep hole, with the top of the hole at the base of the furrow, was excavated with a 30-cm diam. gasoline-powered auger. In-row spacing between plants was 76 cm with rows spaced 86 cm apart. To establish treatments, uninfested field soil was thoroughly mixed with pre-measured inoculum soil to produce the desired level of eggs/g soil for that *Pi*. *Globodera ellingtonae* inoculum soil with average egg densities of 219, 117, and 320 eggs/g soil and 259, 114, and 245 eggs/cyst were used in 2013, 2014, and 2015, respectively. A total volume of ~9,000 cm^3^ of mixed soil (with or without *G. ellingtonae* inoculum) was returned to augured holes. Cut tuber pieces, ~56 g, treated with mancozeb (Maxim MZ; Syngenta Crop Protection, Greensboro, NC) were then placed on top of the soil in the furrow. The area surrounding the experimental area was planted to Russet Burbank for a total of 16 rows. After hand planting, the tubers were treated with imidacloprid (Alias 4F; Adama, Aventura, FL) at 0.44 liter/ha and azoxystrobin (Quadris; Syngenta Crop Protection) at 0.9 liter/ha applied in a 15-cm band over the seed, and then the furrows were closed with additional soil using a tractor-mounted disk hiller. Potatoes were planted May 20, 2013, May 9, 2014, and May 14, 2015. The trial area was sprinkler irrigated and managed with cultural practices common in central Oregon. To control early blight (*Alternaria solani*), plants were treated with boscalid (Endura; BASF, Florham Park, NJ) at 0.33 liter/ha and azoxystrobin at 0.88 liter/ha based upon a growing degree day model used in the region (Gent and Schwartz, 2003). Weeds were additionally managed post-plant with pendimethalin (Prowl; BASF) at 2.34 liter/ha. Potatoes were monitored weekly for vigor including stunting and chlorosis.

Potatoes were harvested approximately 18-weeks-after planting. Aboveground biomass was collected 2 wk prior to harvest by clipping stems at surface level. Dry weights were determined after drying in a 50°C oven for at least a week. At harvest, tubers from each plant were hand-dug and individually weighed. At the same time tubers were collected, an approximately 500 g soil sample was collected near the root zone of each plant and placed in plastic bags. Soil samples were air dried on trays in a greenhouse for at least 1 wk and cysts extracted and enumerated as described above.

A one-way analysis of variance (ANOVA) was used to test for differences in mean yields between, inoculum levels in Trials 1, 2, 3, and 5 followed by a Tukey’s HSD test. To test for a relationship between *Pi* and yield, linear regression with log_10_ (*Pi* + 1) was performed. Linear regressions were performed separately for Trials 1, 2, 4, and 5. A Welch two sample *t*-test was used to compare differences in mean yields between treatments for Trial 4. Host status and reproductive ability were measured by RF and *Pf* with differences among *Pi* densities tested by ANOVA and Tukey’s HSD. Data were log_10_ (* x* + 1) when necessary to meet the assumptions of ANOVA. All statistics were performed using JMP 13.

## Results

### Microplot experiments

**Figure 1:**
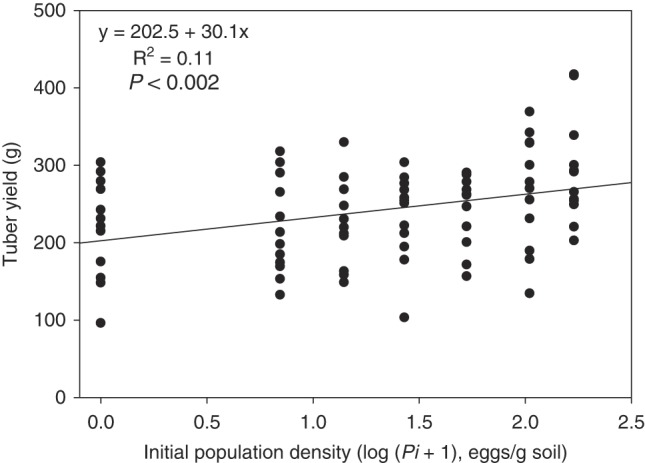
Impact of initial egg density (*Pi*) of *Globodera ellingtonae* on yield of potato (*Solanum tuberosum*) cv. Russet Burbank grown in microplots. A linear model of the relationship between *G. ellingtonae* log_10_ (*P*_*i*_ + 1) and tuber yield was determined.

Simple linear regression revealed that there was a positive correlation between log*Pi* and tuber yield (Fig. 1); this correlation was significant (*P* ≤ 0.002). There was also a positive correlation between log*Pi* and aboveground biomass (*P* ≤ 0.011, *R* ^2^ = 0.08) (data not shown). The number of tubers produced per plant and the average individual tuber weight were not affected by increasing *Pi* (*P* ≥ 0.25) (data not shown). Number of tubers produced per plant ranged from 5 to 9 and average individual tuber weight ranged from 31.3 to 48.7 g.

No cysts were recovered from the non-inoculated control in either trial (data not shown). *Pf* increased with increasing *Pi* (Table 2). While there was a significant difference in the number of *G. ellingtonae* eggs/cyst, for most *Pi* a similar number of eggs/cyst were produced (Table 2). RF values were significantly lower with higher *Pi* compared to lower *Pi* (Table 2).

**Table 2.** Reproduction of *Globodera ellingtonae* inoculated at varying initial population densities (*Pi*) on potato (*Solanum tuberosum*) cv. Russet Burbank in microplots.

*Pi* ^* a*^	*Pf* ^* b*^	Eggs/cyst	RF^c^
6	110 c^d^	165 ab	18.3 a
13	189 bc	200 a	14.5 ab
26	269 ab	162 ab	10.3 bc
52	308 ab	140 ab	5.9 cd
104	376 ab	134 b	3.6 cd
169	410 a	144 ab	2.4 d
*P-value*	≤ 0.001	0.045	≤ 0.001

^a^Initial population densities (Pi) are eggs/g soil; ^b^final population densities (Pf) are eggs/g soil; ^c^RF = final egg density/initial egg density; ^d^values are means of 12 observations. Means within a column followed by the same letter are not different according to Tukey’s honestly significant difference test (P ≤ 0.05).

### Field experiments

**Figure 2:**
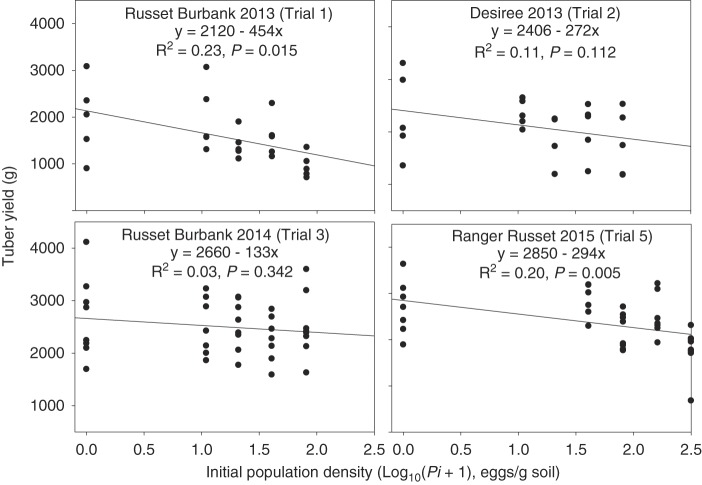
Impact of initial egg density (*Pi*) of *Globodera ellingtonae* on per plant tuber yield of potato (*Solanum tuberosum*) cvs. Russet Burbank, Ranger Russet, and Désirée during three years of field experiments. Separate linear models of the relationship between *G. ellingtonae* log_10_ (*P*_*i*_ + 1) and tuber yield were determined for each trial.

Tuber yields of Russet Burbank averaged 1,580 (±129) and 2,505 (±108) g/plant across all *Pi*s in Trial 1 and Trial 3, respectively. The average tuber yield of Désirée in Trial 2 was 2,087 (±112) g/plant and of Ranger Russet in Trial 5 was 2,347 (±94) g/plant. Average per plant tuber yields for Russet Burbank were significantly different between Trial 1 and Trial 3, and between Désirée (Trial 2) and Russet Burbank (Trial 5) (*P* ≤ 0.01). Simple linear regression revealed that there was a negative correlation between log*Pi* and tuber yield for Trials 1, 2, 3, and 5 (Fig. 2); however, the negative correlation was only significant for Russet Burbank in Trial 1 (*P* ≤ 0.015) and Ranger Russet in Trial 5 (*P* ≤ 0.005). In all of the experiments, a *Pi* of 80 eggs/g soil was included (Table 3). For the cultivar trial (Trial 4), no significant differences were observed between yields of inoculated (*Pi* = 80 eggs/g soil) and non-inoculated plants for any of the potato cultivars (*P* > 0.05) (Table 3). The only difference between yield of inoculated (*Pi* = 80 eggs/g soil) and non-inoculated plants was observed for Russet Burbank in Trial 1 and Ranger Russet in Trial 5 (*P* ≤ 0.05); there was a 43 and 19% reduction in yield of potato when *G. ellingtonae* was present compared to when the nematode was not present, respectively (Table 3). Comparing the *Pf* from the 80 eggs/g soil treatment among the potato cultivars (Trial 4) revealed significantly higher *Pf* for Russet Burbank than for three cultivars: Alturas, Umatilla Russet, and Yukon Gold (*P* ≤ 0.05) (Table 3). Likewise, the same significant differences between Russet Burbank and those three cultivars were observed for RF values (*P* ≤ 0.001). As another indicator of host quality, we compared the mean final eggs/cyst between all cultivars. All cultivars had significantly fewer eggs/cyst than Russet Burbank inoculated with 80 eggs/g soil (Table 3).

**Table 3.** Yield of potato cultivars inoculated (80 eggs/g soil) or not (0 eggs/g soil) with *Globodera ellingtonae* and reproductive outcomes of *G. ellingtonae* when inoculated at an initial population density of 80 eggs/g soil at Powell Butte, Oregon.

	Potato yield (g)				
Cultivar (Trial)	0 eggs/g soil	80 eggs/g soil	*Pf* ^a^	Eggs/cyst	RF^b^
Russet Burbank (Trial 1)	1,878^c^	1,063*^d^	194	119	1.6
Désireé (Trial 2)	2,329	1,781	230	229	3.3
Russet Burbank (Trial 3)	2,677	2,516	292 b^e^	331 b	3.7 b
Alturas (Trial 4)	3,071	3,178	103 a	198 a	1.3 a
Ranger Russet (Trial 4)	2,610	2,428	122 ab	203 a	1.5 ab
Russet Norkotah (Trial 4)	1,777	1,691	127 ab	200 a	1.6 ab
Umatilla Russet (Trial 4)	2,494	2,760	97 a	157 a	1.2 a
Yukon Gold (Trial 4)	1,257	1,560	40 a	170 a	0.5 a
Ranger Russet (Trial 5)	2,701	2,181*	195	220	2.4

^a^Final population densities (Pf) are eggs/g soil; ^b^RF = final egg density/initial egg density; ^c^values are the mean of seven observations; ^d*^denotes a significant difference in yield of inoculated (80 eggs/g soil) compared to non-inoculated (0 eggs/g soil) plants (P < 0.01); ^e^means in a column followed by the same letter for Trials 3 and 4 are not significantly different according to Tukey’s honestly significant difference test (P < 0.05).

Final egg densities (*Pf*) tended to increase with increasing *Pi* except for Russet Burbank in Trial 1, where the highest *Pf* was observed at *Pi* = 20 eggs/g soil (Table 4). Although, in general, higher *Pf* were observed at higher *P*_*i*_, the highest RF values generally occurred at the lowest *Pi* (Table 4). This trend was significant (*P* ≤ 0.05) in all of the field trials except for Russet Burbank in Trial 3. In all of the other years/cultivars the lowest *Pi* (10 or 40 eggs/g soil) had RF values at least five times greater than the corresponding highest *Pi* (80 or 320 eggs/g soil). There was some variability in egg/cyst across the *Pi* in Russet Burbank in Trial 3 and Ranger Russet in Trial 5. In general, there were fewer eggs/cyst at the highest *Pi* in these trials compared to at least one of the lower *Pi* (Table 4).

**Table 4.** Reproduction of *Globodera ellingtonae* inoculated at varying initial population densities (*Pi* ) on potato (*Solanum tuberosum*) cultivars grown in field experiments at Powell Butte, Oregon.

	Russet Burbank (Trial 1)			Désireé (Trial 2)			Russet Burbank (Trial 3)			Ranger Russet (Trial 5)		
	*Pf* ^b^	Eggs/cyst	RF^c^	*Pf*	Eggs/cyst	RF	*Pf*	Eggs/cyst	RF	*Pf*	Eggs/cyst	RF
10	132 abd	287 ab	13.2 a	197	289	19.8 a	50 b	343	5.0			
20	274 a	374 a	13.7 a	213	267	10.7 ab	67 b	244	3.4			
40	294 ab	281 ab	7.3 a	330	247	8.3 b	283 a	313	7.1	159	273 a	4.0 a
80	119 b	195 b	1.5 b	230	229	2.8 c	292 a	331	3.7	195	220 ab	2.4 a
160										169	201 ab	1.1 b
320										209	167 b	0.7 b
*P*-value	0.009	0.03	<0.001	0.10	0.23	<0.001	<0.001	0.20	0.21	0.62	0.008	<0.001

^a^Initial population densities (Pi) are eggs/g soil; ^b^final population densities (Pf) are eggs/g soil; ^c^RF=final egg density/initial egg density; ^d^ values are means of six observations. Means within a column followed by the same letter are not different according to Tukey’s honestly significant difference test (P ≤ 0.05).

## Discussion

This is the first experimental evidence regarding potential yield loss of potato caused by *G. ellingtonae*. High yield losses of potato caused by other species of PCN have been reported for decades, with as much as 80% yield loss at high nematode densities (Seinhorst, 1965; Brown, 1969; Greco and Moreno, 1992). Several studies report losses ranging from 25 to > 50% at *Pi* of 40 eggs/g soil and from 35 to 75% at *Pi* of 80 eggs/g soil, nematode densities similar to those tested in this study (Seinhorst, 1982; Elston et al., 1991; Greco and Moreno, 1992; Van den Berg et al., 2006). Similar yield loss to those reported for PCN might be expected for *G. ellingtonae* based on its phylogenetic proximity to PCN and high reproductive values on potato (Zasada et al., 2013); however, this level of loss due to *G. ellingtonae* was not consistently observed in two microplot and five field experiments conducted in Oregon.

Experiments with PCN have led to several proposed models to describe the relationship between *Pi* and potato yield (Seinhorst, 1965, 1982; Oostenbrink, 1966; Brown, 1969; Elston et al., 1991). These yield loss models vary greatly in their complexity, the more sophisticated of which include additional parameters such as minimum yield and damage thresholds. Separate experiments are required to determine those parameters for a new species at a new location, the experimental design for which would require far more inoculum than we were able to generate in the few years since the discovery of *G. ellingtonae* and the start of our research program with this nematode. Working within the constraints of our inoculum supply, we used mid-range initial nematode densities, shown both to be linearly correlated with yield and to have large negative effects on yield by PCN (Mulder et al., 1997), to determine if similar densities of *G. ellingtonae* would have a detectable effect.

As stated in the introduction, initially *G. ellingtonae* was found at very low densities (1-7 cysts/2.3 kg soil) across the farm at Powell Butte, OR, with one hotspot being identified with 24 cysts/2.3 kg soil. Therefore, two experimental approaches were undertaken to evaluate the pathogenic effects of *G. ellingtonae*. In the field experiments, we chose to utilize an artificial inoculation strategy. Similar to other studies, we inoculated the root zone with plant-parasitic nematodes and assessed plant growth in relationship to *Pi* (Thomas and Clark, 1983; Lamondia, 1995) We recognize the inoculation approach used could result in an underestimation of effect compared to a study in which the *Pi* was distributed over a larger volume of soil. Given that most root proliferation initially occurs near the plant (Kotcon et al., 1984; Opena and Porter, 1999) and the majority of *G. ellingtonae* eggs exposed to potato root diffusate hatch within seven days of exposure (Zasada et al., 2013), we expect our localized application of inoculum produced results similar to that observed with more distributed inoculum. In a study on the root distribution of potato in an irrigated field setting, the majority of the roots were found in the upper 20 cm of soil, with 3.9 cm roots/cm^3^ soil, compared to deeper in the soil profile down to 40 cm, with 0.9 cm roots/cm^3^ soil (Lesczynski and Tanner, 1976). In that study, horizontal distribution of potato roots at a depth of 10 to 20 cm increased with distance from the plant up to 25 cm from the plant. To further ensure we were encompassing potential pathogenic effects of *G. ellingtonae* on potato, we also conducted the microplot trials where the entire soil profile in which potato roots grew contained nematodes.

Field trials 1 and 5 were conducted with increasing *Pi* levels on Russet Burbank and Ranger Russet, respectively. Both trials showed a significant negative correlation between log*Pi* and tuber yield. However, the linear models explained a notably small amount of the tuber weight variability (*R* ^2^ = 0.23 for Russet Burbank and *R* ^2^ = 0.20 for Ranger Russet). Based on the linear regression model of tuber yield on log*Pi* for Trial 1, 30.5 to 40.9% yield loss was predicted at a *Pi* of 40 and 80 eggs/g soil, respectively, and for Trial 5, 16.5 and 19.7% yield loss was predicted at a *Pi* of 40 and 80 eggs/g soil. In the three other field trials and in the microplot trials, *G. ellingtonae* did not negatively affect tuber yield or plant growth. Additionally, no plant health issues (chlorosis, stunting, delayed germination) were observed on the potato plants in either the field or microplot experiments.

We already have reported that host resistance to *G. ellingtonae* varies with potato cultivar, generally in a pattern similar to *G. rostochiensis* (Zasada et al., 2013; Whitworth et al., 2018). However, host tolerance does not always directly correspond with host resistance (Trudgill, 1991). After observing a significant negative correlation between *Pi* and yield for Russet Burbank but no significant effect on Désirée in 2013, a trial was added in 2014 with different cultivars of potatoes to investigate whether maturity class might interact with effects on yield. Although some have found a correlation between maturity class and tolerance, others have not (Trudgill and Cotes, 1983; Arntzen and Wouters, 1994). None of the cultivars tested had significantly reduced yields compared to non-inoculated plants when inoculated with *Pi* of 80 *G. ellingtonae* eggs/g soil. Of the cultivars tested in the field, the host status of Désirée, Modoc, Umatilla, Russet Burbank, and Yukon Gold for *G. ellingtonae* had previously been evaluated in the greenhouse (Zasada et al., 2013). Consistent with greenhouse results, Yukon Gold had a lower, although not significantly so, RF value in the field than Russet Burbank, Umatilla, and Modoc. *Globodera ellingtonae* had higher *Pf* on Russet Burbank than Alturis, Umatilla Russet and Yukon Gold and higher eggs/cyst on Russet Burbank than all other six cultivars, consistent with previous observations (Zasada et al., 2013). This is notable as Russet Burbank is the most widely planted cultivar in the USA (National Agricultural Statistics Service, 2018).

Final egg densities in the field trials had maximum means ranging from 273 to 330 when inoculated with *Pi* of 40 eggs/g soil. In the microplot trials, *Pf* reached maximum means of 357 (*Pi* = 52) and 328 (*Pi* = 169) eggs/g soil. Combined, these results indicate successful invasion and reproduction of *G. ellingtonae* in all trials. Studies of PCN reproduction indicate the maximum *Pf*, often in a range of 300 to 400 eggs/g soil, usually occurs at intermediate *Pi* (~32–50 eggs/g soil) and that *Pf* values either level out or even decline with increasing *Pi* (Phillips et al., 1991; Greco and Moreno, 1992; Ehwaeti et al., 2000). The *Pi* of *G. ellingtonae* at which the maximum *Pf* occurred differed between years and between trials conducted within a year. As seen with PCN (Trudgill et al., 2014), RF values generally decreased with increasing *Pi* in our trials; the Russet Burbank 2014 trial was the only exception.

Field studies of PCN using the same potato cultivar for multiple years have documented that the rate of yield reduction varies with year, with effects of year sometimes eclipsing effects of *Pi* (Mulder et al., 1997). Our results are consistent with that pattern, with the difference in Russet Burbank yield between years 2013 and 2014 being much greater than the effect of *G. ellingtonae* in any given year. Although some studies have determined that relative yield loss caused by PCN increases with increasing total yield (Mulder et al., 1997; Phillips et al., 1998), we did not find such a correlation given that the only significant reduction in Russet Burbank yield was seen in the trial with a lower mean yield.

The pathogenic effect of *G. ellingtonae* on yield of potato was inconsistent across years and experimental venues. Five field trials were conducted over a three-year period and of these there was a significant negative relationship between *G. ellingtonae Pi* and tuber yield in the Russet Burbank Trial 1 and the Ranger Russet Trial 5. In two microplot experiments, there were no negative effects of varying *G. ellingtonae Pi* on tuber yield. Given that a field study comparing all three *Globodera* spp. at a single site is not feasible, using these data to compare pathogenic effects between species is not possible. Great care should be taken in extrapolating the results to probable effects in other environments, as effects of PCN on given potato cultivars vary when grown at different sites (Dale et al., 1988; Phillips et al., 1998). To date, *G. ellingtonae* has been reported from morphological descriptions only in Oregon and Idaho (USA) and Argentina, with presence in Chile inferred from molecular data (Skantar et al., 2011; Handoo et al., 2012; Lax et al., 2014). It will be of interest to determine the pathogenic effects of *G. ellingtonae* to potato in its presumably native environments of South America.
